# Breast cancer: age incidence, hormone receptor status and family history in Najaf, Iraq

**DOI:** 10.25122/jml-2022-0296

**Published:** 2022-10

**Authors:** Hasan Khalaf, Alaa Mohammed, Sinan Shukur, Noor Alhalabi, Basim Almothafar, Mustafa Hassan, Abduelraheim Abu

**Affiliations:** 1 Department of General Surgery, Kufa Medical College, University of Kufa, Kufa, Iraq; 2 Department of Surgery, Dentistry College, Islamic University, Najaf, Iraq; 3 Department of Medicine, Faculty of Medicine, University of Kufa, Kufa, Iraq; 4 School of Medicine, University of Jordan, Amman, Jordan; 5 Faculty of Medicine, University of Kordofan, El Obeid, Sudan

**Keywords:** breast cancer, family history, hormone receptor status

## Abstract

Breast cancer is the primary type of cancer affecting women. Patients with hormone receptor-positive cells have lower mortality rates. Both chemotherapy and hormone therapy can improve the survival rate. This study aimed to evaluate the hormonal receptor status in female breast cancer and assess the relationship with the patient's age and family history in Najaf, Iraq. A prospective study of two-hundred and fifty-one women with mastectomies (for cancers) was performed at AL Sader Medical City from January 2019 to January 2021. We collected and analyzed data regarding the age of patients, site and size of the tumor, number of pregnancies, family history, weight, smoking, and hormone receptor status. The average age of patients was 48 years, and the peak incidence was in the 40–49 age group (30.27% of patients). Of all age groups, 48.6% (122 patients) were negative for all hormone receptors (triple negative), and only 22.70% had a positive family history. The peak incidence of cancer in our study was in the 40–49 years group. A high proportion of the hormone receptors for patients were negative (triple-negative), and most patients had a negative family history.

## INTRODUCTION

Breast cancer is one of the top causes of cancer-related deaths in women worldwide and is thought to be the most frequent female malignancy [1, 2]. However, the incidence among multicultural populations varies, suggesting that etiologic factors vary in their biological expression and influence the course of the disease. According to research, the number of breast cancer-related cases in Iraq increased from 26.6/100,000 in 2000 to 31.5/100,000 in 2009 [3]. In 2012, 11,833 new cancer cases were registered in women [4]. The terminal ductless with sac-like ends (alveoli), which make up the primary secretory units of the breast, are grouped together and form the breast lobules by being encased in a thin, specialized connective tissue. Progesterone is required for lobular formation, while estrogen is essential for ductal system development. It is now widely accepted that genetic and epigenetic changes that result in abnormal growth regulation and disruption of intracellular signaling frequently cause breast cancer to begin in the epithelium that lines the terminal ductless within the lobule. As a result, breast cancer is regarded as a heterogeneous disease with numerous subtypes and cells with unique cellular origins and functions [5]. The most prevalent type of breast cancer is invasive or infiltrating duct carcinoma (IDC), while the most prevalent histological form of the non-invasive (in situ cancer) stage of breast cancer is ductal carcinoma in situ (DCIS). Features of the tumor and the status of the estrogen receptor (ER), progesterone receptor, and human epidermal growth factor receptor 2 (HER2) in the tumor, affect the treatment of breast cancer [6]. Five biological subtypes have been found for breast cancer based on the expression of ER, PR, HER2 receptors, and nuclear antigen Ki-67 (Table 1) [7]. The steroid hormone expression, together with HER2 receptors, form the crucial prognostic factor and the predictive factor of breast cancer outcome [8].

**Table 1:** Biological breast cancer subtypes.

Subtypes of breast cancer	Estrogen receptor	Progesterone receptor	HER2 receptor	Ki-67
Luminal A-like	+ve	+ve	-ve	Low (>20%)
Luminal B-like (HER2 negative)	+ve	-ve or lower	-ve	High (<20%)
Luminal B-like (HER2 positive)	+ve	Any	+ve	Any
HER2 positive (HER2 enriched)	-ve	-ve	+ve	Any
Triple-negative (basal-like)	-ve	-ve	-ve	Any

ER and PR form the vast majority of breast cancers. According to the literature, breast tumors with high steroid hormone receptor expression are less aggressive and have a better prognosis. At the same time, the triple-negative subtype that accounts for 15–25% of cancer cases has the worst prognosis, linked with a poor prognosis, aggressive histology, and resistance to the standard endocrine therapy [9]. It should be highlighted that when evaluated by suitable immune-histochemical methods, only about 85% of triple-negative phenotypic breast tumors are basal-like [10, 11]. Estrogen receptor (ER) and progesterone receptor (PR) are nuclear receptors measured in breast tumor specimens for prognostication of disease recurrence and prediction of treatment response [12]. Estrogen receptor positive will assist in predicting the outcome of endocrine therapy, such as the administration of antiestrogen (tamoxifen) or ovarian suppression. Human epidermal growth factor receptor 2 (HER2) is associated with high-grade breast cancer and a high risk of recurrence; its assessment is important for targeting therapy with monoclonal antibody (trastuzumab) against HER2. HER-2 expression and the presence of hormone receptors are crucial prognostic indicators in breast cancer [13]. The aim of this study was to evaluate the hormonal receptors status in female breast cancer in relation to the patient's age and family history in Najaf, Iraq.

## Material and methods

Prospective data from 251 female patients with histopathological evidence of breast cancer from AL Sadr Medical City, Najaf, Iraq, were included in the study. AL Sadr Medical City is a university tertiary hospital with 1500 beds situated in the center of the city, serving two million people from Najaf city in the middle of Iraq. This study included patients who were referred to the breast clinic at AL Sadr Medical City, Najaf, Iraq, from January 2019 to January 2021, complaining of breast mass and with positive FNA for malignancy. Breast and axillary ultrasounds were performed for the primary tumor staging, and file systems for recording all patient data were organized. All patients underwent a mastectomy, and the biopsies were sent for histopathological study to confirm the diagnosis, staging, and tumor grading. We analyzed data regarding the patient's age, site, size of the tumor, number of pregnancies, family history, weight, smoking, hormone receptor status, histological staging, and age of menarche. Based on the status of their ER (estrogen receptor), PR (progesterone receptor), and HER2 (human epidermal growth factor) receptors, each patient was categorized into one of eight (ER/PR/HER2) groups.

The clinical five-level categories of 0, I, II, III, and IV were used to transform data from the tumor, nodes, and metastasis (TNM) stage at diagnosis. PR status was noted according to the pathologist's interpretation of the assays. Immunoperoxidase levels under 5% are deemed negative for ER and PR. Analyzing cytosol protein can also reveal the ER and PR status staining of tumor cell nuclei. When complete and intense membrane staining was found in more than 10% of tumor cells, HER-2/neu overexpression was deemed positive. Benign breast tumors and secondary breast cancer from other primary cancer other than the breast were excluded from the study. Results were collected, analyzed, and compared using an interactive calculator (chi-square). The p-value of <0.05 was regarded as significant.

## Results

### Patients' age

Table 2 shows that the age of patients ranged from 20 to over 80 years and that the average age was 48 years. The peak incidence was in the 40–49 age group (30.27% of patients), followed by the 50–59 (22.7%) and the 30–39 age group (21.5%). The least affected age group was the ≥70 group (4.78%).

**Table 2:** Distribution of patients according to age.

Age	No. of patients	%
20–29	14	5.57
30–39	54	21.51
40–49	76	30.27
50–59	57	22.7
60–69	38	15.13
≥70	12	4.78
Total	251	100%

### Age-specific distribution of hormone receptors

Table 3 shows the distribution of hormone receptors among age groups. The hormone receptors under investigation were ER, PR, and HER2. The distribution of hormone receptors was divided into eight groups, ranging from all positive to all negative. Of all age groups, 48.6% (122 patients) were negative for all HR (triple-negative). Most participants with triple-negative were in the ≥70 age group (58.34%), followed by the 60–69 age group (55.26%), 20–29 (50.0%), 50–59 (49.10%), 40–49 (48.68%) and 30–39 (40.74%) respectively. Only 7 patients showed all HR-positive status, 2.78% of the sample, and it was more common (two patients, 5.26%) in the age group 60–69 than in other groups. None of the patients in the age group 20–29 and ≥70 had all HR- positive simultaneously. Only 9.16% (23 of patients) had ER+ and PR +only. Most of them (15.78%) were in the age group 60–69, and none in the ≥70 had ER & PR positive only. 17.13% (43) had only HER2 positive; the peak percentage of 25% was in the age group ≥70 (Table 3).

**Table 3:** Relationship between hormone receptors and age groups.

Age	E-, P+, H-		E+, P-, H+		E+, P+, H+		E+, P+, H-		E+, P-, H-		E-, P-, H-		E-, P-, H+		E-, P+, H+		Total	
	No	%	No	%	No	%	No	%	No	%	No	%	No	%	No	%	No	%
**20–29**	2	14.28	0	0%	0	0%	1	7.14	0	0%	7	50.0	3	21.42	1	7.14	14	100%
**30–39**	9	16.67	2	3.7	1	1.85	5	9.25	3	5.56	22	40.74	12	22.23	0	0%	54	100%
**40–49**	10	13.15	1	1.31	3	3.94	6	7.89	4	5.26	37	48.68	12	15.78	3	3.94	76	100%
**50–59**	6	10.52	2	3.5	1	1.75	5	8.77	3	5.26	28	49.10	9	15.78	3	5.26	57	100%
**60–69**	1	2.63	0	0%	2	5.26	6	15.78	4	10.52	21	55.26	4	10.52	0	0%	38	100%
**70–more**	0	0%	0	0%	0	0%	0	0%	1	8.34	7	58.34	3	25%	1	8.34	12	100%
**Total**	28	11.15	5	1.99	7	2.78	23	9.16	15	5.97	122	48.60	43	17.13	8	3.18	251	100%

E – estrogen hormone receptor; P – progesterone hormone; H – human epidermal growth factor; No – number of patients.

### Distribution of family history

Table 4 shows the distribution of family history according to the age of the patients, with only 22.70% of the patients showing positive family history. Positive family history was more prevalent in the 20–29 age range (50%) but less common in the old age group: 2.63% in the age group 60–69 and 8.33% in the age group ≥70.

**Table 4:** Relationship of cancer with family history.

Age	Family H+	%	Family H-	%	Total	%
20–29	7	50%	7	50%	14	100%
30–39	17	31.48	37	68.52	54	100%
40–49	22	28.94	54	71.05	76	100%
50–59	9	15.78	48	84.21	57	100%
60–69	1	2.63	37	97.36	38	100%
70–more	1	8.33	11	91.67	21	100%
Total	57	22.70	194	77.30	251	100%

P=0.004; Family H+ – positive family history of breast cancer; Family H- – negative family history of breast cancer.

## Discussion

Cancer is the second most common cancer in the world; the prevalence rates range almost four times between different parts of the world, from 27 per 100,000 in Middle Africa and Eastern Asia to 96 in Western Europe [14]. Breast cancer at a young age is associated with an aggressive type of cancer. In our study, the peak incidence of cancer was in the 40–49 age group (30.27% of total patients), and this rate is correlated with another two studies from Iraq at 30.3% [15] and 31.9% [16]. The peak of age incidence in this study correlates with another statistic from Indonesia [17]. It was found that Iraq's age-related incidence rate was higher than that of Turkey, Iran, Saudi Arabia, and Bahrain but lower than that of Jordan and Kuwait [14]. In the United States, only 19% of patients with breast cancer were younger than 50 years between 2017 and 2018 [18]. They found that Iraq has the highest breast cancer risk of any nation, which may be related to the sociopolitical situation in the nation (chemical warfare, bombings etc). This is perhaps why southern governorates like Basra and Thi-Qar have a greater breast cancer risk [15]. In comparison to statistics from other countries, most of them show peak age incidence older than the Iraqi one: India (50–64 years) 2005 [19], China (55–60 years) 2017, Sweden 45–54 years, 2019 [20], Vietnam 45–55 years, 2015 [17], Singapore 50 years, 2007 [17], Malaysia, 52 years, 2010 [17], Australia 65–69 years, 2008 [17], USA over 60 years, [21, 22], Tanzania 36–55 years, 2013 [23]. That is to say, breast cancer in Iraq affects middle-aged women more than other age groups. In our research, we noticed a large percentage of patients with triple-negative hormone receptors (48.60%); this was most pronounced in young women (20–29 years), where it was 50.0% and premenopausal period (middle age groups) (48.68%). This indicates that most patients in our study had hormonally negative receptors. Those tumors show a high-grade, highly proliferative, metastatic disease that does not respond to established endocrine therapy with poor outcomes [23–25]. Although our result correlates with a study from Tanzania by 45.6% and India [22], compared to breast cancer cases recorded in other parts of the world, the majority of instances were positive hormone receptors. In Canada, it was 11.2% [26], UK 16.3% [27], China 17% [28], Egypt 19.0% [29], and Malaysia 17.6% [30]. One of the reasons that could be behind the triple-negative is the younger age at presentation among Iraqi women. Reduced exposure to exogenous estrogens, such as those found in oral contraceptives and hormone replacement therapy, is another important factor influencing this shift in the proportion of cases. These external hormones lead to a higher occurrence of hormone receptor-positive tumors compared to negative hormone receptors in cancer cases. In previous Indian (Huang WY) studies, it was analyzed and mentioned that breast cancer caused by non-hormonal causes produces hormonal receptor-negative. The number of patients with a positive family history (57 patients), 22.70%, was lower than patients with negative family history (194 patients), 77.30%. Positive family history and young women with breast cancer are significantly correlated. Positive family history is higher in young patients 20–29 (50%) and 30–39 (31.48%) groups. In this study, a positive family history of breast cancer similarly increases the risk for positive and negative hormonal receptors tumors. The increased risk associated with a positive family history may reflect many inherited factors. These factors may lead to ER-positive tumors or ER-negative tumors. Identifying families (multiple affected members whose tumors show concordant receptor expression) may allow the clarification of specific implementations that differentiate positive from negative receptor cancers [31].

## Conclusion

The peak incidence of cancer in this study was in the 40–49 age group. In comparison to other statistics from other countries, the majority had a peak age incidence older than the one identified in Iraq. Also, we found a significant percentage of patients triple negative for hormone receptors, which was most obviously seen in young women. The majority of patients had a negative family history.
